# 肺硬化性血管瘤的诊治

**DOI:** 10.3779/j.issn.1009-3419.2011.08.07

**Published:** 2011-08-20

**Authors:** 少华 马, 宇 孙, 长征 杜, 震 梁, 宏超 熊, 克能 陈

**Affiliations:** 100142 北京，北京大学肿瘤医院暨恶性肿瘤发病机制及转化研究教育部重点实验室胸外一科 Key Laboratory of Carcinogenesis and Translational Research (Ministry of Education), Department of Thoracic Surgery I, Peking University School of Oncology, Beijing Cancer Hospital and Institute, Beijing 100142, China

**Keywords:** 肺硬化性血管瘤, 手术, 病理, Pulmonary sclerosing hemangioma, Surgery, Pathology

## Abstract

**背景与目的:**

肺硬化性血管瘤(pulmonary sclerosing hemangioma, PSH)概念的提出迄今只有50余年，是一种少见的肺部良性疾病。PSH临床表现有一定特点，需与肺癌鉴别诊断。本文总结我院48例PSH的临床诊治经验并复习文献，旨在提高对PSH的认识，探讨合理的诊断与治疗手段。

**方法:**

2001年1月-2011年4月共收治PSH 48例，结合文献报道总结分析PSH的发病特点、临床表现、影像学、病理学特点及预后。

**结果:**

全组48例，无症状者27例(56.3%)，肿物大小0.2 cm-7.0 cm，平均2.1 cm，各个肺叶均有发生。合并肺门或纵隔淋巴结肿大者15例(31.3%)。手术47例，其中肺叶部分切除29例(61.7%)，肺叶切除14例(29.8%)，肿物剔除3例(6.4%)，前纵隔肿物切除者1例(2.1%)。47例术后均无复发。CT引导下穿刺活检诊断1例，随访28个月未见肿瘤进展。

**结论:**

PSH术前定性诊断困难，手术既是确诊手段又是有效的治疗手段，该病预后良好。

## Abstract (en)

**Background and objective:**

The concept of pulmonary sclerosing hemangioma (PSH) was proposed only 50 years ago. PSH features several characteristics that should be differently diagnosed with lung cancer. The aim of this study is to retrospectively review the diagnosis and treatment of 48 cases of PSH, review recent publications about this topic, and address reasonable diagnosis and treatment methods.

**Methods:**

Forty-eight patients with PSH were enrolled and consecutively treated from January 2001 to April 2011. Clinical presentations, image characteristics, pathologic morphologies, and prognostic data were analyzed.

**Results:**

Twenty-seven PSH cases (56.3%) were asymptomatic. Tumor diameters ranged from 0.2 to 7.0 cm, with an average diameter of 2.1 cm, and involved both lobes of the lungs. Enlarged lymph nodes in the mediastinum were detected via computed tomography (CT) scans in 15 cases (31.3%). Only 47 patients underwent surgery. Pulmonary wedge resection was performed in 29 cases (61.7%) while lobectomy was performed in 14 (29.8%). The tumor was removed in 3 cases (6.4%) and anterior mediastinal tumor resection was performed in 1 case (2.1%). Forty-seven patients reported no recurrence during follow-up while one patient refused surgery after a CT-guided biopsy with no progression during the 28-month follow up period.

**Conclusions:**

The exact diagnosis of PSH is difficult prior to surgery. Surgery is an effective method of treatment that allows both definite diagnosis and accurate therapy. The prognosis of PSH is favorable.

肺硬化性血管瘤（pulmonary sclerosing hemangioma, PSH）的概念1956年由Liebow和Hubbell提出^[1]^。1999年WHO疾病分类将其归为肺杂类良性肿瘤^[2]^，但关于其组织来源、称谓、定义及有价值的免疫组化标记物的争论从未停止过^[3]^。PSH容易出现误诊，值得深入探讨。本文报道北京大学肿瘤医院2001年1月-2011年4月收治的48例PSH病例的诊治经验并结合文献复习进行总结。

## 1 资料与方法

对2001年1月-2011年4月北京大学肿瘤医院收治的经病理确诊的48例PSH的临床表现、影像资料、手术资料、病理切片、免疫组化、转归及随访结果作详细分析。48例PSH中包括男性5例，女性43例，男女之比为1:11.6。诊断年龄23岁-74岁，中位诊断年龄48岁。吸烟者6例，非吸烟者4 2例。无症状体检发现者2 7例(56.3%)，有症状者21例(43.8%)，包括咳嗽11例，血痰5例，胸痛5例，临床表现无特殊。

## 2 结果

### 2.1 影像学表现

全组均行胸片及CT检查，3例行正电子发射断层扫描(position-emission tomography, PET)检查。CT表现为单发者47例，2个病灶者1例；发生于左肺者18例(37.5%)，右肺29例(60.4%)，诊断为前纵隔肿瘤者1例(2.1%)；15例合并肺门或纵隔淋巴结肿大；CT形态表现为圆形或卵圆形肿块，边缘光滑，轮廓清楚，密度基本均匀，少数有点状钙化，增强扫描显示病灶呈中等以上均匀或不均匀强化(图 1)。3例行PET者中1例显示^18^F-FDG高代谢灶(SUV 3.9)(图 2)。所有CT发现的肿物被术后病理证实为PSH，切除标本肿物直径0.2 cm-7.0 cm，平均直径2.1 cm。

**1:**
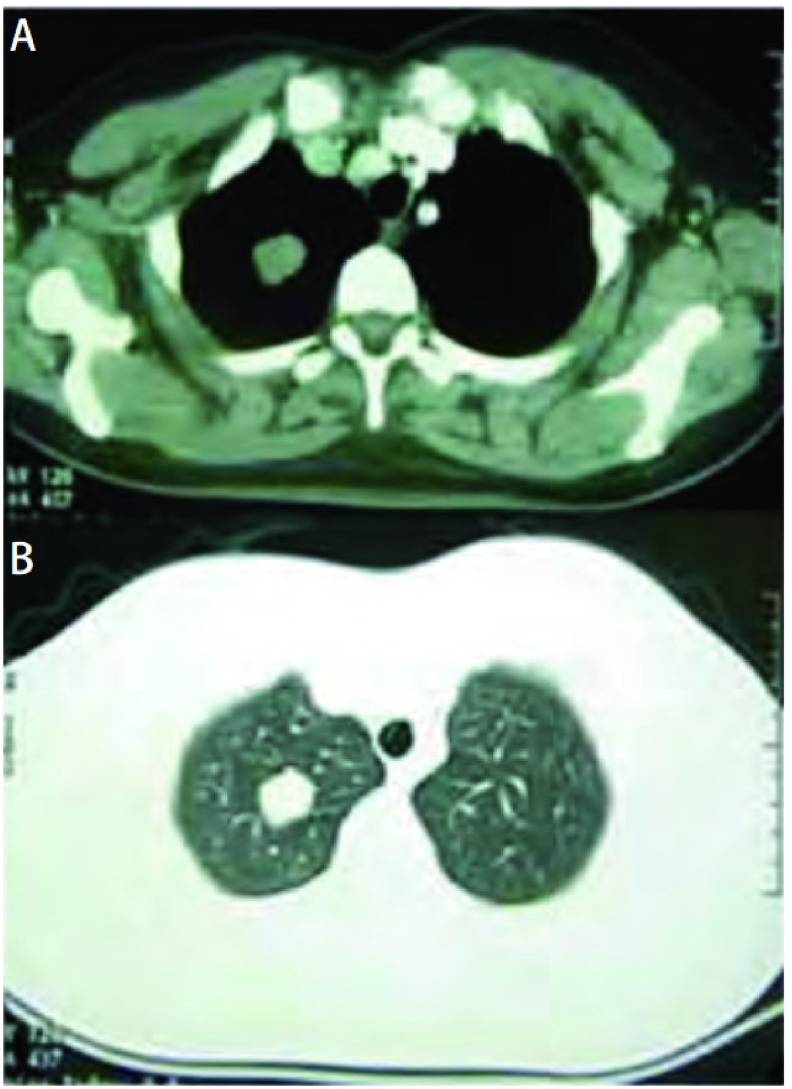
患者女性，35岁，体检发现右肺上叶肿物。CT示类圆形实性肿物，大小为20 mm×17 mm，密度均匀，边缘基本光滑，其内可见钙化斑。A：纵隔窗；B：肺窗。 Female patient, 35 years old, tumor was founded in right upper lobe of lung by medical examination. CT shows 20 mm×17 mm spherical solid mass, uniform density, boundary smooth and calcification within. A: mediastinal window; B: lung window.

**2:**
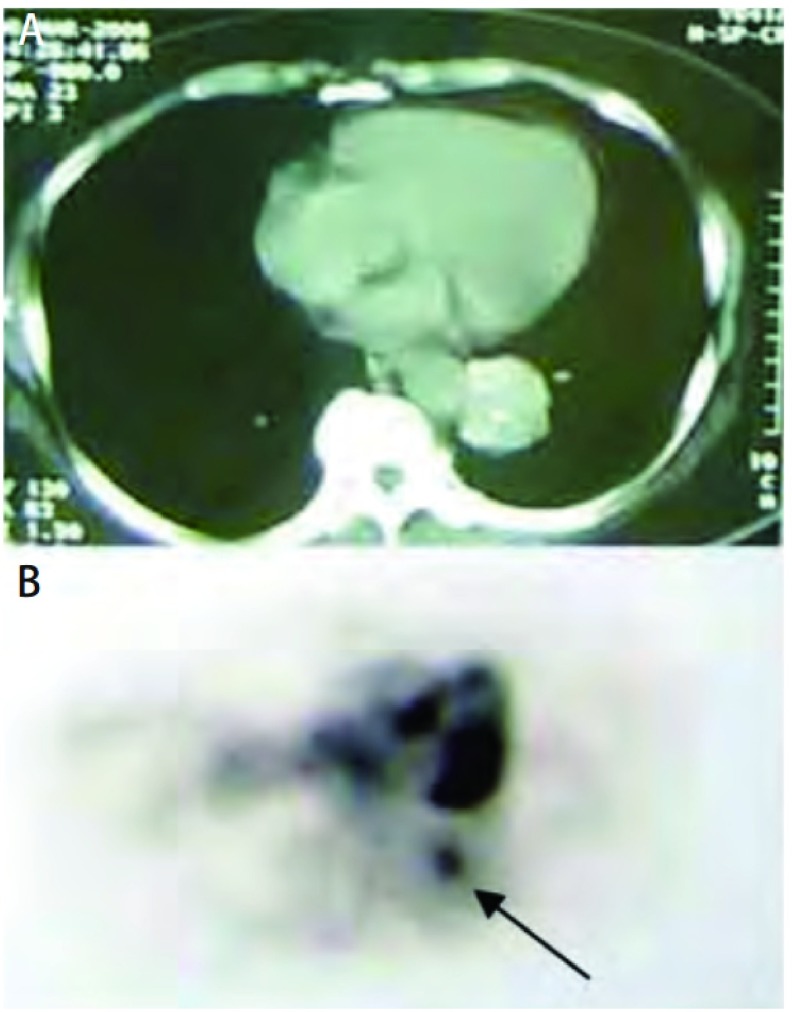
患者女性，74岁，慢性咳嗽。A：CT示左肺下叶35 mm×30 mm肿物，边界欠清，其内可见钙化；B：PET示高代谢灶(如箭头所示)。 Female patient, 74 years old, with chronic cough. A: CT shows left lower lobe 35 mm×30 mm mass, boundary is less clear and calcification; B: PET shows hypermetabolic lesion (arrow).

### 2.2 手术及预后

全组48例除1例拒绝手术外，余47例均予手术治疗。其中肺叶部分切除29例(61.7%)，肺叶切除14例(29.8%)；肿物剔除3例(6.4%)，按前纵膈肿物切除1例(2.1%)。47例术后均顺利康复，未见术后并发症。疑为肺癌行纵隔淋巴结清扫15例，送检淋巴结均呈反应性增生，未见转移。随访0.5个月-104个月，中位随访时间为32个月，失访6例。随访期间47例均未见复发、转移及死于该疾病。1例患者穿刺活检确诊后拒绝手术，随诊28个月病变无进展。

### 2.3 病理形态学

48例经HE染色可见到典型的PSH形态学表现，即乳头状结构、实性区、出血区及硬化性改变，由立方上皮细胞和圆形的间质细胞组成(图 3)。免疫组化检查中8例行甲状腺转录因子-1(thyroid transcription factor-1, TTF-1)染色，上皮细胞和间质细胞均呈强阳性表达，8例行上皮细胞膜抗原(epithelial membrane antigen, EMA)染色，上皮细胞和间质细胞均呈强阳性表达(图 4)，5例行波形蛋白(Vimentin)染色，间质细胞均呈阳性表达，2例行突触素(Synaptophysin)染色，间质细胞均呈阳性表达。

**3:**
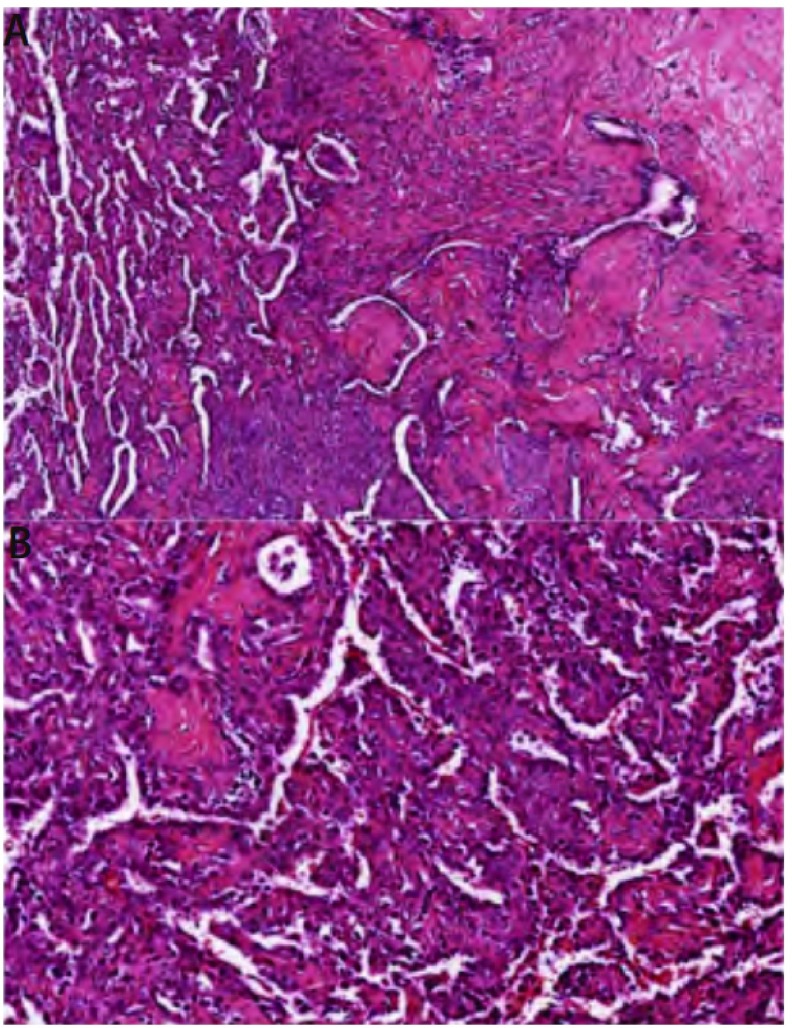
PSH HE染色病理图片。A：左侧可见乳头状结构，复杂的乳头被覆立方上皮细胞，乳头突起的蒂内含间质细胞；右侧为实性结构，间质细胞呈片状分布，有立方上皮细胞形成的小管散在其中；实性区内可见致密的透明变性胶原灶，即硬化区(HE，×100)；B：衬覆于乳头状结构表面的立方上皮细胞和位于乳头状结构轴心的圆形或多角形的间质细胞，瘤细胞形状大小较一致，核浆比例小，核圆形或卵圆形。(HE，×200) PSH HE staining pathological picture. A: The left area shows papillary pattern: complex papillae lined by cuboidal surface cells, the stalk of the papillary projections contains the round stromal cells. The right area shows solid pattern: sheets of round cells, with scattered cuboidal surface cells forming small tubules. dense foci of hyaline collagen within the solid areas is sclerotic pattern (HE, × 100); B: Surface cells and round stromal cells are uniform in size and shape, with round to oval nuclei (HE, ×200).

**4:**
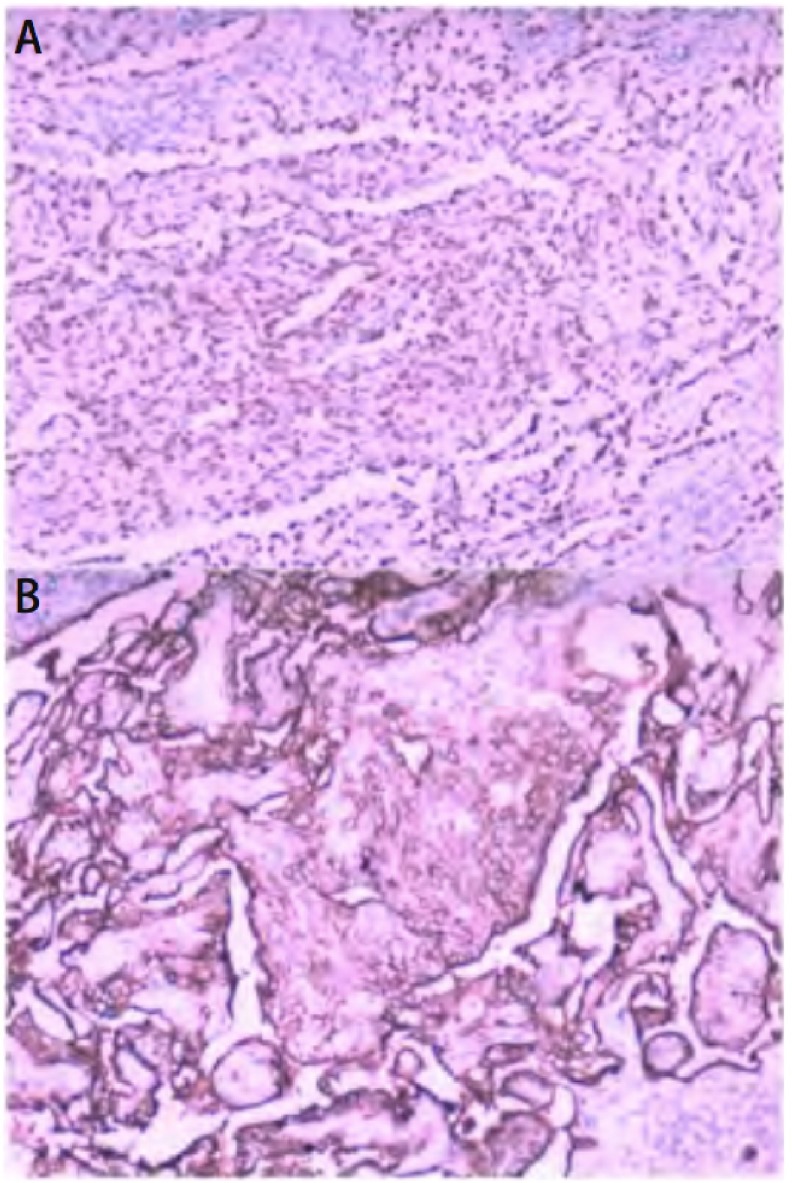
PSH免疫组化病理图片。A：圆形间质细胞和立方上皮细胞核阳性(TTF1阳性，×100)；B：圆形间质细胞和立方上皮细胞膜阳性(EMA阳性，×100)。 PSH immunohistochemical pathology images. A: TTF1 shows nuclear staining of round and surface cells (TTF1 positive, ×100); B: EMA shows membranous staining of round and surface cells (EMA positive, ×100).

## 3 讨论

### 3.1 PSH称谓的历史演变及其组织来源

1965年由Liebow和Hubbell^[1]^提出PSH的概念前全球临床及病理医生均将其归类为"炎性假瘤"，因两者治疗手段相近，故并未受到重视。PSH的称谓系由该肿瘤镜下表现与皮肤的硬化性血管瘤(又称为组织细胞瘤或黄瘤)相似而得名，当时认为系血管来源的肿瘤，并得到一些学者的认可^[4, 5]^，与此同时也有学者认为PSH来源于未分化的肺泡上皮细胞^[6]^。此后，在PSH的电镜检查中未发现血管内皮细胞所特有的WP(Weible-Palade)小体，相反发现了上皮细胞特有的桥粒及微绒毛结构^[7]^，从而使上皮性来源理论占了上风。随着免疫组化的发展，在PSH中找到了更多具有上皮细胞特有的免疫组化标记物如EMA和TTF-1，进一步确立了PSH上皮来源理论。目前，多数学者认为PSH来源于肺上皮细胞^[3, 8]^。1999年WHO将其列为肺杂类少见良性肿瘤，但是鉴于PSH的称谓已经习惯，WHO分类系统仍选用了PSH的条目^[2]^。当然，在PSH演变的过程中，有人还将其诊断为"错构瘤”等其他类型的肿瘤。

### 3.2 临床表现与诊断治疗

PSH好发生于亚裔非吸烟的女性，文献^[8, 9]^报道男女发病之比为1:5，40岁-60岁多见。多数患者无自觉症状，部分患者可出现无诊断特异性的症状，如咳嗽、血痰及胸痛等^[10]^。PSH的CT表现多数为孤立的外周型肿物，偶见双肺多发病灶者(4%-5%)，肿物呈圆形或类圆形，直径多为2 cm-3 cm，边缘光滑，轮廓清楚，密度均匀，内部可见钙化点，增强CT呈中等以上均匀强化(40%)或不均匀强化(60%)^[11]^。文献[^12]^报道“空气新月征”为相对特异的影像学表现，但临床较为少见。PSH可发生肺门及纵隔淋巴结转移，但仅限于个案报道，且多数转移发生在肿瘤直径 > 3.5 cm者^[13]^，也有学者认为多发病变为PSH的肺内转移^[14, 15]^。PSH在PET上可以表现为高代谢灶^[16, 17]^，与恶性肿瘤鉴别诊断价值有限。本组3例患者接受PET检查，其中1例病灶呈现^18^F-FDG异常浓聚，术前误诊为肺癌。针刺活检对确诊PSH有一定帮助，但由于PSH的多形表现，诊断的准确性取决于穿刺区域的细胞形态，有时受到取材量的限制，与高分化腺癌和神经内分泌癌难以鉴别^[3]^。

### 3.3 病理形态表现

由于临床无特殊表现，PSH主要靠病理学确诊。PSH大体形态是一种边界清楚、黄褐色的外周型肿块，直径在0.3 cm-7 cm之间，但也有发生在支气管内或表现为胸膜息肉样肿物^[18]^。PSH在镜下形态学上主要表现为乳头状结构、实性区、出血区及硬化性改变四种组织构型，常以不同比例混合存在^[19]^。PSH由两种细胞成分构成，即衬覆于乳头状结构表面的立方上皮细胞和位于实性细胞区与乳头状结构轴心的圆形或多角形的间质细胞，瘤细胞形状大小较一致，核浆比例小，核类圆形或类圆形，核仁淡染、染色均匀、无明显核分裂象；部分瘤组织中见噬含铁血黄素的单核细胞、泡沫细胞浸润^[8, 13]^。尽管这些特征都体现了PSH在组织学上异质性的特点，但个别情况下与高分化腺癌和神经内分泌癌仍难以鉴别^[3]^。

### 3.4 免疫组化在PSH中的特殊地位

虽然文献^[8, 13, 19]^罗列了很多HE染色的镜下特点，但在1965年以前PSH的标准诊断仍为“炎性假瘤”，而且，目前个别情况下与高分化腺癌和神经内分泌癌仍难以鉴别^[3]^，这说明仅凭HE染色形态学诊断PSH有一定的局限性，需要良好的具有特异性免疫组织化学标记物来进行鉴别诊断。然而以前对此认识并不足够，回顾本组48例，只有8例做了EMA染色，上皮细胞和间质细胞均呈强阳性表达；8例做了TTF-1染色，上皮细胞和间质细胞均呈强阳性表达；5例做了Vimentin染色，间质细胞均呈阳性表达；2例做了突触素染色，间质细胞均呈阳性表达。综上，应该说本组主要的诊断依据仍为HE染色，若按现代对PSH的免疫组化认识再分析，难免有疏漏之处。为此，本文总结了目前常用有鉴别意义的免疫组化标记物于表 1，供大家参考。其中TTF-1和EMA对PSH的诊断价值最为突出^[24]^。

**1:** PSH中的蛋白表达 Protein expression in PSH

Study	EMA			TTF-1			Surf			CAM5.2			SMA			Pan-K			ER			PR	
	SC	RC		SC	RC		SC	RC		SC	RC		SC	RC		SC	RC		SC	RC		SC	RC
Yoo^[20]^	+	+		+	+		n/a	n/a		-	-		n/a	n/a		+	+		-	-		-	-
Nicholson^[21]^	+	+		+	+		-	-		n/a	n/a		-	-		+	-		n/a	n/a		n/a	n/a
Illei^[22]^	+	+		+	+		+	+/-		+/-	+/-		n/a	n/a		n/a	n/a		n/a	n/a		n/a	n/a
Kim^[23]^	+	+		+	+		n/a	n/a		n/a	n/a		-	-		+	-		n/a	n/a		n/a	n/a
Devouassoux-Shisheboran^[8]^	+	+		+	+		+	-		+	+/-		n/a	-		+	-		n/a	+/-		n/a	+
SC: surface cells; RC: round cells; EMA: epithelial membrane antigen; TTF-1: thyroid transcription factor-1; Surf: surfactant apoprotein; CAM5.2: low molecular weight cytokeratin; SMA: smooth muscle actin; Pan-K: pan-keratin; ER: estrogen receptor; PR: progesterone receptor.																							

总之，对PSH的认识是随着多学科的发展呈渐进过程。PSH为良性肿瘤，外科手术是诊断和治疗PSH最有效的手段，预后良好。但仍有许多问题有待解决，临床医师应该做到知识更新，以免出现误诊和漏诊。
